# Effectiveness of glatiramer acetate compared to other multiple sclerosis therapies

**DOI:** 10.1002/brb3.337

**Published:** 2015-05-01

**Authors:** Guillermo Izquierdo, Nuria García-Agua Soler, Macarena Rus, Antonio José García-Ruiz

**Affiliations:** 1 Department of Neurology, Hospital Universitario Virgen MacarenaAvenida Dr. Fedriani, 3, 41009, Seville, Spain; 2 Chair of Health Economics and Rational Drug Use, School of Medicine, University of MálagaBoulevard Louis Pasteur, 32, 29071, Málaga, Spain; 3 Department of Pharmacology and Clinical Therapeutics, School of Medicine, University of MálagaBoulevard Louis Pasteur, 32, 29071, Málaga, Spain

**Keywords:** Clinical practice, effectiveness, glatiramer acetate, multiple sclerosis, therapy, treatment

## Abstract

**Objective:**

To assess the effectiveness of glatiramer acetate (GA) compared to other multiple sclerosis (MS) therapies in routine clinical practice.

**Materials and methods:**

Observational cohort study carried out in MS patients treated with GA (GA cohort) or other MS therapies –switched from GA– (non-GA cohort). Study data were obtained through review of our MS patient database. The primary endpoint was the Expanded Disability Status Scale (EDSS) scores reached at the end of treatment/last check-up.

**Results:**

A total of 180 patients were included: GA cohort *n* = 120, non-GA cohort *n* = 60. Patients in the GA cohort showed better EDSS scores at the end of treatment/last check-up (mean ± SD, 2.8 ± 1.8 vs. 3.9 ± 2.2; *P* = 0.001) and were 1.65 times more likely to show better EDSS scores compared to the non-GA cohort (odds ratio, 0.606; 95%CI, 0.436–0.843; *P* = 0.003). Patients in the GA cohort showed longer mean time to reach EDSS scores of 6 (209.1 [95%CI, 187.6–230.6] vs. 164.3 [95%CI, 137.0–191.6] months; *P* = 0.004) and slower disability progression (hazard ratio, 0.415 [95%CI, 0.286–0.603]; *P* < 0.001). The annualized relapse rate was lower in the GA cohort (mean ± SD, 0.5 ± 0.5 vs. 0.8 ± 0.5; *P* = 0.001) and patients’ quality of life was improved in this study cohort compared to the non-GA cohort (mean ± SD, 0.7 ± 0.1 vs. 0.6 ± 0.2; *P* = 0.01).

**Conclusions:**

GA may slow down the progression of EDSS scores to a greater extent than other MS therapies, as well as achieving a greater reduction in relapses and a greater improvement in patients’ quality of life. Switching from GA to other MS therapies has not proved to entail a better response to treatment.

## Introduction

Multiple sclerosis (MS) is an immune-mediated demyelinating and neurodegenerative disease of the central nervous system mainly diagnosed in young adults, representing the major cause of nontraumatic disability in this segment of population (Koutsouraki et al. 2010). The disease involves a life-long, unpredictable course generally categorized as relapsing-remitting, secondary progressive, and primary progressive, though all these courses entail a progressive destruction of myelin (Richards et al. 2002). As there is no cure available yet, the main goals of therapy are to prevent relapses and slow down neurological deterioration.

Glatiramer acetate (GA) is a widely used disease-modifying drug indicated for the reduction of relapses in patients with relapsing-remitting MS and for the treatment of patients who are at high risk of developing clinically definite MS. GA's mechanism of action has not been fully elucidated yet, but it appears to be related to its immunomodulatory effect and neuroprotective properties (Aharoni et al. 2003; Schrempf and Ziemssen 2007; Blanchette and Neuhaus 2008). The first head-to-head clinical trials addressing its efficacy in comparison with other disease-modifying agents showed similar efficacy between GA and interferon *β* (INF*β*) in patients with relapsing-remitting MS (Mikol et al. 2008; Cadavid et al. 2009; O'Connor et al. 2009), which was supported by a recently published meta-analysis (La Mantia et al. 2014). Conversely, another meta-analysis reported greater efficacy of GA in terms of Kurtzke's Expanded Disability Status Scale (EDSS) scores and clinical progression compared to IFN*β* (Qizilbash et al. 2012). The few studies directly comparing MS therapies used in clinical practice also suggested lower progression indexes in patients treated with GA compared to IFN*β*-1b (Haas and Firzlaff 2005), as well as greater decline in relapse rates (Haas and Firzlaff 2005; Castelli-Haley et al. 2010) and lower risk of relapse in comparison with IFN*β*-1a (Ollendorf et al. 2002; Castelli-Haley et al. 2010) or IFN*β*-1b (Ollendorf et al. 2002). However, there are no data available yet to know whether patients who discontinue GA would have a better response to other MS therapies.

In this study, we compared patients receiving GA with patients who switched from GA to other MS therapies in order to assess whether the effectiveness of other MS therapies after the treatment switch was at least comparable to the effectiveness of GA.

## Materials and Methods

This was a retrospective observational study conducted at Hospital Universitario Virgen Macarena (Seville, Spain) in accordance with the World Medical Association Declaration of Helsinki, all its amendments and national regulations. All patients provided informed consent for treatment with GA or other MS therapies, which were administered from commercial sources according to the technical specifications and clinical practice. This study was approved by the ethics committee of Hospital Universitario Virgen Macarena (Seville, Spain).

### Patient population

The study population comprised all MS patients included in the database of the Department of Neurology at Hospital Universitario Virgen Macarena (Seville, Spain) until October 1st, 2012 who were receiving GA (Copaxone®; Teva Pharmaceuticals Ltd., London, United Kingdom) (GA cohort) and those who switched from GA to other MS therapies due to inadequate effectiveness or adverse events (non-GA cohort). MS was diagnosed according to McDonald criteria (McDonald et al. 2001) and all treatments were administered according to routine clinical practice.

### Assessments

Study data were obtained through review of information contained in the department's MS patient database, which included demographics, clinical MS characteristics at diagnosis and at the beginning of the study treatments (baseline), and MS course during patients’ follow-up until database lock on October 1st, 2012. The following patients’ data were collected: gender, date of birth, MS diagnosis, clinical findings, evoked potentials, results on magnetic resonance imaging and cerebrospinal fluid analysis, study treatments (drugs and treatment durations), MS duration (at baseline and at the end of the study treatment, if that occurred, or last check-up), EDSS scores (at baseline and at the end of the study treatment, if that occurred, or at last check-up), disability progression until database lock, number of relapses during the study treatments, and health-related quality of life (at baseline and at the end of the study treatment, if that occurred, or last check-up).

A relapse was defined as the appearance of a new neurologic symptom or worsening of a preexisting one, not attributable to fever or other concurrent phenomenon, which persisted for more than 24 h and became evident during a neurological examination. Quantification of patients’ disability was performed according to EDSS scores (Kurtzke 1983). Disability progression was defined as an increase in EDSS scores ≥1.0 points for patients with EDSS scores <5.5, or ≥0.5 points for patients with EDSS scores ≥5.5, persisting on a second examination performed 3 months later. Health-related quality of life was assessed according to the EuroQoL 5D (EQ-5D) questionnaire, which consists of a descriptive system of five dimensions (mobility, self-care, usual activities, pain/discomfort, and anxiety/depression) and a visual analog scale (Brooks 1996; Badia et al. 1999). The patients were asked to rate their health state against the most appropriate statement (no problems, some problems, or extreme problems) in each of the five dimensions and on a vertical visual analog scale where the endpoints were marked as 100 (best imaginable health state) and 0 (worst imaginable health state). Summary EQ-5D index values calculated from patients’ rate of their health state may range from 1 (full health) to 0 (death).

### Statistics

The primary efficacy endpoint was the EDSS scores reached at the end of the study treatment/last check-up. Comparison of these scores between patients in the GA and non-GA cohorts was performed using the ANOVA test. A Kaplan–Meier analysis was also performed to assess the proportion of patients according to scores on the EDSS in both cohorts, which were compared using the Cox proportional hazards test.

The secondary efficacy endpoints comprised the time to reach EDSS scores of 4 and 6; the progression of disability until database lock; the maintenance/improvement on EDSS scores at the end of treatment/last check-up; the effect of demographic and clinical variables on the improvement in EDSS scores; and the number of relapses experienced by patients during the study treatment. Kaplan–Meier estimates were used to assess the time to reach EDSS scores of 4 and 6 in the GA and non-GA cohorts, which were compared using log-rank tests. Patients’ disability progression in both cohorts was assessed by the Kaplan–Meier method and compared using the Cox proportional hazards test. Descriptive analyses were performed to assess the proportion of patients with maintained/improved EDSS scores in the study cohorts, which were compared using the Pearson's chi-squared test. A multivariate logistic regression analysis was also performed to evaluate the effect of independent variables (i.e., age at diagnosis, age when starting the study treatment, gender, time from the diagnosis of multiple sclerosis to the beginning of treatment, GA treatment, EQ-5D indexes at the beginning of the study treatment, EDSS scores at the beginning of the study treatment, EDSS scores at the end of the study treatment/last check-up, duration of the study treatment, clinical findings, evoked potentials, and results on magnetic resonance imaging and cerebrospinal fluid analysis) on the improvement/maintenance of EDSS scores. Descriptive analyses were used to assess the number of relapses experienced by patients in each study cohort, which were compared using the ANOVA test. Another secondary endpoint included the effect of the study treatments on patients’ health-related quality of life, which was assessed according to scores on the EQ-5D questionnaire. Summary EQ-5D indexes were calculated from EQ-5D scores at the end of treatment/last check-up in the study cohorts, which were compared using the ANOVA test.

A propensity score matching was also performed to make sure that potential selection bias did not affect the study outcomes in the GA and non-GA cohorts (i.e., EDSS scores at the end of the study treatment/last check-up, the time to reach EDSS scores of 4 or 6, the proportion of patients that maintained/improved EDSS scores at the end of the study treatment/last check-up, the annualized relapse rate and the EQ-5D index at the end of treatment/last check-up). The variables included in the multivariate logistic regression analysis were also included in the propensity score matching (i.e., age at diagnosis, age when starting the study treatment, gender, time from the diagnosis of multiple sclerosis to the beginning of treatment, GA treatment, EQ-5D indexes at the beginning of the study treatment, EDSS scores at the beginning of the study treatment, EDSS scores at the end of the study treatment/last check-up, improved EDSS scores at the end of the study treatment/last check-up, duration of the study treatment, clinical findings, evoked potentials, and results on magnetic resonance imaging and cerebrospinal fluid analysis). All covariates were balanced for both cohorts and the nearest-neighbor matching was used.

Missing data were not considered in the analyses and a significance level of 0.05 was used for statistical testing. The statistical analyses were performed with the Statistical Package for the Social Sciences (SPSS) version 20.0 (SPSS Inc., Chicago, IL).

## Results

### Patient characteristics

A total of 180 patients were included in the study, 120 of whom comprised the GA cohort and 60 comprised the non-GA cohort. Baseline demographic and clinical characteristics are described in Table 1.

**Table 1:** Baseline demographic and clinical characteristics of patients.

Patient characteristics	GA cohort (*N* = 120)	Non-GA cohort (*N* = 60)	*P*-value
Age (years), mean ± SD			
At baseline	44.5 ± 10.6	40.7 ± 10.2^1^	0.025
At MS diagnosis	35.4 ± 10.9^2^	31.4 ± 8.7^3^	0.023
Female, *n* (%)	76 (63.3)	45 (76.3)^1^	0.082
MS course *n* (%)			
RRMS	112 (93.3)	52 (86.7)	0.138
SPMS	8 (6.7)	8 (13.3)	
Baseline EDSS scores, mean ± SD	2.0 ± 1.3^4^	2.4 ± 1.7^4^	0.133
Number of relapses in the year previous to starting the study treatment, mean ± SD	0.97 ± 0.18	0.98 ± 0.13	0.521
Baseline EQ-5D index values, mean ± SD	0.76 ± 0.11	0.73 ± 0.14	0.133
MS length at baseline (months), mean ± SD	61.2 ± 74.3^4^	67.3 ± 83.6^4^	0.626

EDSS, Kurtzke's Expanded Disability Status Scale; EQ-5D, EuroQoL 5D; GA, glatiramer acetate; MS, multiple sclerosis; RRMS, relapsing-remitting multiple sclerosis; SD, standard deviation; SPMS, secondary progressive multiple sclerosis.

1 Missing data, *n* = 1

2 Missing data, *n* = 15

3 Missing data, *n* = 10; ^4^Missing data, *n* = 4.

The duration of the study treatments did not significantly differ between the GA and non-GA cohorts (mean ± SD, 81.1 ± 51.8 vs. 92.4 ± 60.2 months; *P* = 0.305; Table 2). In the latter, the main MS treatment was interferon beta-1a (23 [38.8%] patients), followed by interferon beta-1b (11 [17.7%] patients), fingolimod (10 [16.5%] patients), natalizumab (10 [15.9%] patients), mitoxantrone (4 [6.5%] patients), and other therapies such as alemtuzumab, amantadine, azatioprine, and corticosteroids (3 [4.7%] patients).

**Table 2:** Summary of patients’ clinical characteristics at the end of the study treatment/last check-up.

Patient characteristics	GA cohort (*N* = 120)	Non-GA cohort (*N* = 60)	*P*-value
EDSS scores at the end of the study treatment/last check-up, mean ± SD	2.8 ± 1.8^1^	3.9 ± 2.2^1^	0.001
Number of relapses during the year previous to the end of the study treatment/last check-up, mean ± SD	0.1 ± 0.3	0.3 ± 0.5	<0.001
Annualized relapse rate during the study treatments, mean ± SD	0.5 ± 0.5	0.8 ± 0.5	0.001
Total number of relapses during the study treatments, mean ± SD	4.8 ± 3.1	8.3 ± 4.6^2^	<0.001
EQ-5D index values at the end of the study treatment/last check-up, mean ± SD	0.7 ± 0.1^1^	0.6 ± 0.2^1^	0.001
Duration of study treatments (months), mean ± SD	81.1 ± 51.8^1^	92.4 ± 60.2^1^	0.305

EDSS, Kurtzke's Expanded Disability Status Scale; EQ-5D, EuroQoL 5D; GA, glatiramer acetate; SD, standard deviation.

1 Missing data, *n* = 4

2 Missing data, *n* = 1.

### Effectiveness

**Figure 1:**
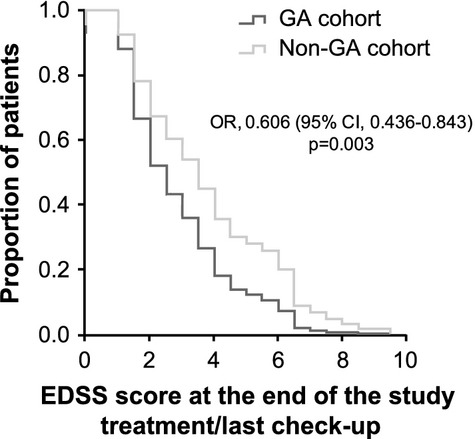
Proportion of patients according to Expanded Disability Status Scale scores. CI, confidence interval; EDSS, Kurtzke's Expanded Disability Status Scale; GA, glatiramer acetate; OR, odds ratio.

**Figure 2:**
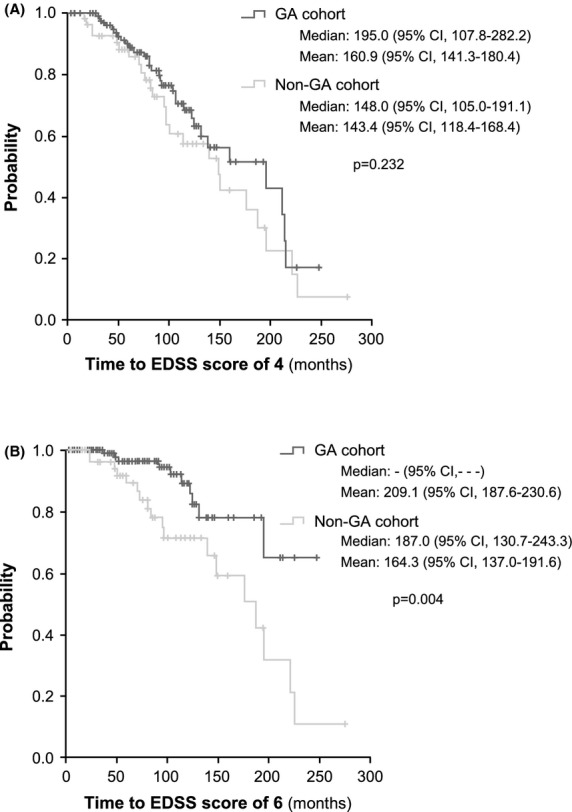
Time to Expanded Disability Status Scale scores of 4 (A) and 6 (B). CI, confidence interval; EDSS, Kurtzke's Expanded Disability Status Scale; GA, glatiramer acetate.

**Figure 3:**
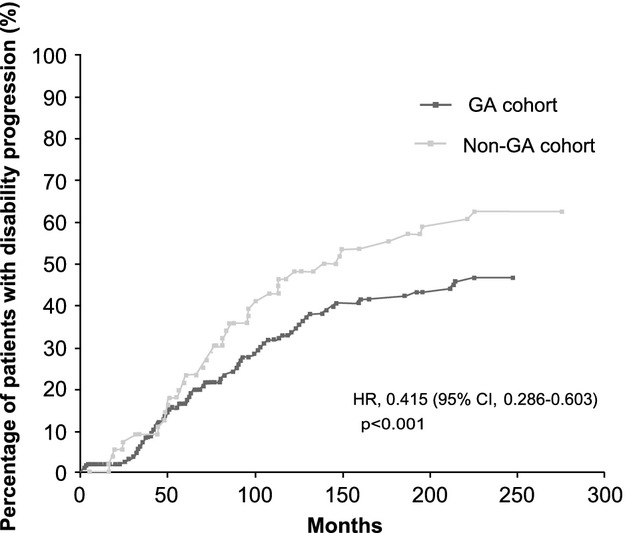
Percentage of patients with disability progression according to Expanded Disability Status Scale scores. CI, confidence interval; HR, hazard ratio; GA, glatiramer acetate.

Even though there were no statistically significant differences between the study cohorts either in the EDSS scores at the beginning of the study treatments (Table 2003) or in their treatment duration (Table 1999), patients in the GA cohort showed better EDSS scores at the end of the study treatment/last check-up in comparison with those receiving other MS therapies (mean ± SD, 2.8 ± 1.8 vs. 3.9 ± 2.2; *P* = 0.001; Table 1999). Indeed, patients in the GA cohort were 1.65 times more likely to show better EDSS scores at the end of treatment/last check-up than patients from the non-GA cohort (odds ratio [OR], 0.606; 95%CI, 0.436–0.843; *P* = 0.003; Fig.1); for each patient treated with other therapy, there were 65% more patients with better EDSS scores in the GA cohort. The mean time to reach EDSS scores of 4 varied from 160.9 months (95%CI, 141.3–180.4 months) in the GA cohort to 143.4 months (95%CI, 118.4–168.4 months) in the non-GA cohort, with median times of 195.0 months (95%CI, 107.8–282.2 months), and 148.0 months (95%CI, 105.0–191.1 months) (*P* = 0.232), respectively (Fig.2A). When the cut-off was raised to an EDSS score of 6, statistically significant differences were found between both cohorts of patients, showing that the mean time to reach this EDSS score was 209.1 months (95%CI, 187.6–230.6 months) in patients treated with GA, whereas it was 164.3 months (95%CI, 137.0–191.6 months) in those receiving other therapies (*P* = 0.004) (Fig.2B). Additionally, disability progression was significantly slower in the GA cohort compared to the non-GA cohort (hazard ratio [HR], 0.415; 95%CI, 0.286–0.603; *P* < 0.001; Fig.3) and the proportion of patients that maintained/improved EDSS scores at the end of the study treatment/last check-up tended to be higher among patients receiving GA (53.5% vs. 37.5%; *P* = 0.05). The results of the multivariate analysis performed to assess the effect of independent variables on the improvement/maintenance of EDSS scores only showed statistically significant differences according to GA treatment (OR, 2.600; 95%CI, 1.226–5.514; *P* = 0.013) and initial EDSS score (OR, 1.436; 95%CI, 1.117–1.847; *P* = 0.005).

**Figure 4:**
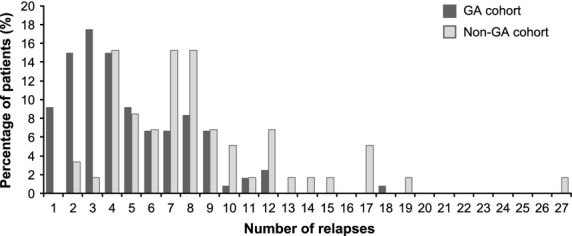
Number of relapses during the study treatment. GA, glatiramer acetate.

The annualized relapse rate during the study treatment was significantly lower in the GA cohort than in the non-GA cohort (mean ± SD, 0.5 ± 0.5 vs. 0.8 ± 0.5; *P* = 0.001), as well as the number of relapses reported in the year previous to the end of the study treatment/last check-up (mean ± SD, 0.1 ± 0.3 vs. 0.3 ± 0.5; *P* < 0.001) (Table 1999). Likewise, the total number of relapses during the study treatment was significantly lower in patients receiving GA compared to those not receiving it, with a mean (±SD) number of 4.8 ± 3.1 and 8.3 ± 4.6 relapses (*P* < 0.001), respectively (Table 1999). In the GA cohort, 11 (9.2%) patients reported just one relapse, 68 (56.7%) between two and five relapses, and 41 (34.2%) more than five relapses, whereas no patient in the non-GA cohort reported having experienced only one relapse, 17 (28.8%) patients between two and five relapses, and 42 (71.2%) more than five relapses (Fig.4).

Propensity score matching did not affect the results obtained on EDSS scores at the end of the study treatment/last check-up, the time to reach EDSS scores of 4 or 6, the proportion of patients that maintained/improved EDSS scores at the end of the study treatment/last check-up or the annualized relapse rate.

### Health-related quality of life

Statistically significant differences in EQ-5D index values were found between the GA and non-GA cohorts at the end of the study treatment/last check-up, showing improved quality of life in those patients under GA treatment (mean ± SD, 0.7 ± 0.1 vs. 0.6 ± 0.2; *P* = 0.01; Table 1999). Differences between both cohorts of patients were also shown in the proportion of patients with EQ-5D indexes of 1.0–0.8, 0.8–0.6, 0.6–0.4, 0.4–0.2, and 0.2–0.0 (*P* = 0.017), with a higher percentage of patients in the GA cohort with EQ-5D indexes of 1.0–0.8 (37 [31.9%] vs. 14 [25.0%] patients) and 0.8–0.6 (48 [41.4%] vs. 17 [30.4%] patients).

After the propensity score matching, the differences observed in EQ-5D index values between the study cohorts at the end of treatment/last check-up remained significant.

## Discussion

This study shows that patients in the GA cohort maintained better EDSS scores at the end of the study treatment/last check-up than those in the non-GA cohort and were 1.65 times more likely to show better EDSS scores than patients in the non-GA cohort. Patients in the GA cohort also showed longer time to reach EDSS scores of 6, slower disability progression, and a trend toward higher proportion of patients maintaining/improving EDSS scores at the end of treatment/last check-up. Differences in disability progression between MS therapies used in clinical practice are controversial, as no significant differences have been reported among intramuscular INF*β*-1a, subcutaneous INF*β*-1a, subcutaneous INF*β*-1b and GA in terms of changes in EDSS scores and the percentage of progression-free patients (Haas and Firzlaff 2005). However, despite not reaching statistical significance, the EDSS scores were lower and the percentage of progression-free patients was higher when GA was used compared with other IFN*β* formulations (Haas and Firzlaff 2005). Additionally, the 2-year treatment period assessed in this study might have been too short to detect differences in these parameters and the calculation of progression index still showed improvements when GA was used in comparison with subcutaneous INF*β*-1b (Haas and Firzlaff 2005). The results obtained from head-to-head clinical trials also showed the absence of significant differences in short-term disability progression between 2-year treatment with GA, subcutaneous INF*β*-1a, and subcutaneous INF*β*-1b (Mikol et al. 2008; O'Connor et al. 2009; La Mantia et al. 2014), though the percentage of patients with progression was lower in those receiving GA compared to subcutaneous INF*β*-1a (Mikol et al. 2008). Although GA has not evidenced greater short-term effect on reducing disability progression compared to INF*β*, its long-term activity may enable brain atrophy to be prevented, as shown by the reduced number of black holes observed in magnetic resonance imaging (Rovaris et al. 2007). Our results suggest an improved control of disability progression in patients under a longer treatment with GA in routine clinical practice, which is in line with the results obtained from a meta-analysis that found a significant reduction in the risk of clinical progression in patients being treated with GA in comparison with INF*β* (Qizilbash et al. 2012). However, differences in the design and strength of evidence provided by the different studies should be considered when interpreting our results.

The number of relapses during the study treatment was lower when GA was used in comparison with other MS therapies. Though statistical significance was not shown between GA and INF*β* preparations in the reduction of relapses in head-to-head trials (Mikol et al. 2008; Cadavid et al. 2009; O'Connor et al. 2009; La Mantia et al. 2014), the results achieved by the CombiRx trial supports the greater efficacy of GA in reducing the risk of exacerbations (Lublin et al. 2013). Similarly, the results obtained from observational studies agree with those observed in ours. Indeed, the comparison of the effectiveness of these therapies in clinical practice have even shown a lower risk of relapse with GA in comparison with INF*β*-1a (Ollendorf et al. 2002; Castelli-Haley et al. 2010) and INF*β*-1b (Ollendorf et al. 2002), as well as a greater decline in relapse rates with GA compared to INF*β* formulations (Haas and Firzlaff 2005). In a recent paper of the MSBase Study Group, the cohort of patients treated with subcutaneous IFN***β***-1a or GA were at slightly but significantly lower risk of MS relapses than those treated with intramuscular IFN***β***-1a or IFN***β***-1b, as shown by the annualized relapse rates and the proportions of relapse-free patients (Kalincik et al. 2014).

MS adversely affects patients’ quality of life, mainly as a result of disease-related factors such as disability, fatigue, and depression (Zwibel 2009). However, the quality of life of our patients was shown to be improved when GA was used compared to other MS therapies. Similarly, improvements in patients’ quality of life with MS therapies were previously reported (Lily et al. 2006). Nonetheless, the existence of differences among the different MS therapies has not been completely clarified, as controversial information has been published ranging from the absence of differences among IFN*β*-1a, IFN*β*-1b, and GA (Lily et al. 2006), to detecting a better effect of GA treatment in patients’ quality of life in comparison with IFN*β* preparations (de Seze et al. 2012). Additionally, the improvement we observed agree with the results obtained from the few studies assessing the effect of MS therapies in factors associated with quality of life. Indeed, a greater decrease in fatigue was shown in patients under GA treatment compared to those receiving INF-*β* (Metz et al. 2004), as well as in patients switching from INF-*β* to GA (Hadjimichael et al. 2008). Furthermore, while INF-*β* may be associated with increasing risk of depression in susceptible individuals or aggravating preexisting depression (Mohr et al. 1999; Goeb et al. 2006), there is no evidence that GA aggravates depression (Ziemssen 2009). Indeed, GA may have a beneficial effect on depression as a result of increasing central brain-derived neutrophic factor, stimulating neurogenesis or through its anti-inflammatory effect (Tsai 2007). However, little information directly comparing the effect of MS therapies is currently available and the different questionnaires or scales used considerably increase the difficulty of comparing their results.

The authors acknowledge that several limitations should be considered when interpreting our results, including those derived from the observational study design. Although observational studies provide valuable and more generalizable information regarding the administration of treatments in routine clinical practice, their observational nature precludes achieving strong evidence. Potential biases derived from the collection of data from a preexisting database cannot be ruled out. However, a propensity score matching was performed in order to minimize bias derived from confounding variables that might be found in the study cohorts. Additionally, patients in the non-GA group were treated with different MS therapies, which did not enable head-to-head comparisons to be performed, and no information was collected on the safety profile of the study treatments, which precludes risk-benefit analysis being performed. Though it is difficult to generalize results from a single-site study, the profile of patients described in our study resembles the general population of patients with MS regularly seen in neurology departments. Therefore, the study results should be interpreted with caution; however, we believe that they are meaningful for physicians to manage MS in daily practice.

In conclusion, GA may slow down the progression of EDSS scores in a greater extent than other MS therapies; indeed, patients continuing on GA showed a slower disability progression and a longer time to reach EDSS scores of 6 than patients switching from GA to other MS therapies. Additionally, our study showed a greater reduction in the number of relapses and a greater improvement on patients’ quality of life during the GA treatment. Although it can be hypothesized that patients switching from GA to other MS therapies due to inadequate effectiveness or adverse events might show a better or, at least, comparable response than those whose GA treatment was maintained, our results do not support this hypothesis; even when switching from glatiramer acetate to other multiple sclerosis therapy is justified, it may not end up enabling disease to be controlled. This fact led us to think that these patients may show an overall poor response to any MS therapies (either GA or other MS therapies) and may explain that our findings are not in accordance with those of previous head-to-head trials. Nonetheless, further prospective studies are needed to validate our findings, as well as to provide clinicians with conclusive data regarding the head-to-head comparison of the effectiveness of the different MS therapies available in clinical practice and their impact on patients’ health-related quality of life.
